# A mechanism for ramified rolling circle amplification

**DOI:** 10.1186/1471-2199-11-94

**Published:** 2010-12-07

**Authors:** Thomas P Beals, James H Smith, Raymond M Nietupski, David J Lane

**Affiliations:** 1 Thorne Diagnostics, Beverly, MA, USA

## Abstract

**Background:**

Amplification of single-stranded DNA circles has wide utility for a variety of applications. The two-primer ramified rolling circle amplification (RAM) reaction provides exponential DNA amplification under isothermal conditions, creating a regular laddered series of double-stranded DNA products. However, the molecular mechanism of the RAM reaction remains unexplained.

**Results:**

A RAM reaction model predicts exponential accumulation of a double-stranded DNA product size series, and product-size ratios, that are consistent with observed RAM reaction products. The mechanism involves generation of a series of increasing size intermediate templates; those templates produce RAM products and recursively generate smaller intermediate templates. The model allows prediction of the number of rounds of circular template replication. Real-time RAM reaction data are consistent with the model. Analysis of RAM reaction products shows exponential growth limitation consistent with the model's predictions.

**Conclusions:**

The model provides a rationale for the observed products of the RAM reaction, and the molecular yield among those products. Experimental results are consistent with the model.

## Background

A variety of isothermal nucleic acid amplification technologies (reviewed in [1]) are now available as complements or alternatives to the polymerase chain reaction (PCR). Detection of circularized single-stranded DNA (ssDNA) molecules (padlock probes, [2]) has been brought to bear on applications as diverse as SNP detection [3-5], miRNA detection [6], and molecular diagnostics [7,8]. Isothermal ssDNA circle amplification has been performed by single-primer initiated rolling circle amplification (RCA) and by two-primer amplification methods variously called ramification amplification (RAM, [9]); hyperbranched RCA [10], cascade RCA [11], or exponential RCA [5].

In contrast to single primer RCA [12] and the PCR, no detailed and predictive model for the RAM reaction has yet appeared, although illustrative diagrams of two-primer ssDNA circle amplification have been published [11,13]. Here, we present a model of the RAM reaction that accounts for the size and number of double-stranded DNA (dsDNA) product molecules.

## Results and Discussion

### RAM reaction products and components

**Figure 1:**
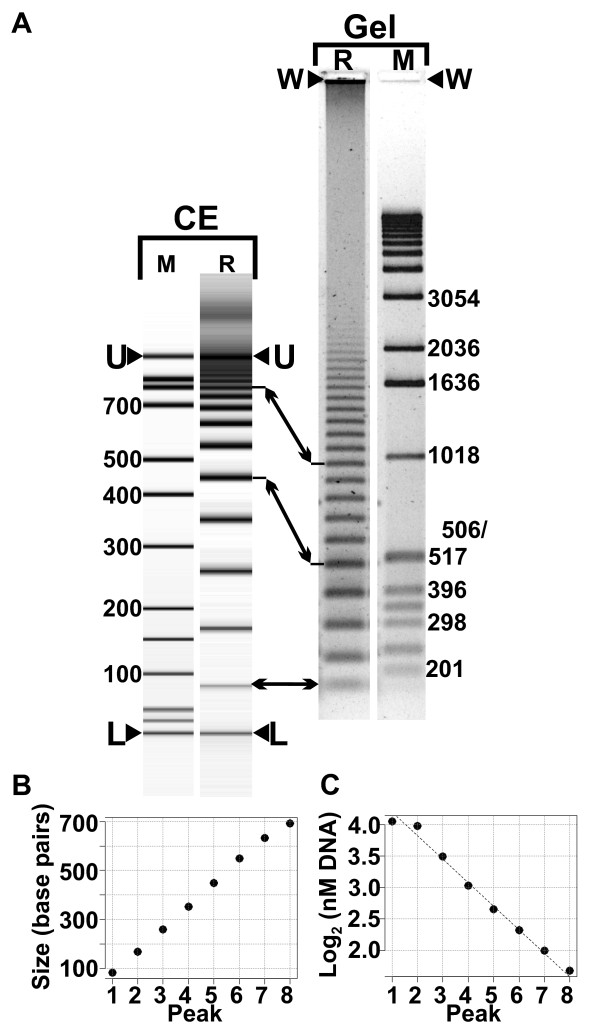
**RAM reaction products**. A) Visualization of RAM reaction products by capillary electrophoresis (CE) and by agarose gel electrophoresis (Gel). CE lane M: CE ladder; CE lane "R", RAM reaction products. Arrowheads labelled U and L indicate CE upper and lower markers. Gel lane R: RAM reaction products (same reaction as CE lane R). Gel lane M, DNA size markers. Arrowheads labelled "W" mark the gels' loading well. Arrows between CE lane R and Gel lane R mark corresponding RAM product bands. B) A plot of DNA band-size vs. band number. C) A plot of log2 (molecule number as nM concentration) vs. band number.

Electrophoresis of RAM reaction products results in a characteristic dsDNA ladder (Figure 1A). Visualization and analysis of the ladder bands shows that the bands' size-increment is equal to the template-circle's circumference (Figure 1B). The initial 8 to 10 bands appear to be of approximately equal fluorescence intensity. Equal fluorescence with increasing size suggests a decrease in molecule-number that is proportional to the length of the DNA molecule in the band. Figure 1C shows a log-linear relation between molecule number and product size; the following model accounts for those observations.

**Figure 2:**
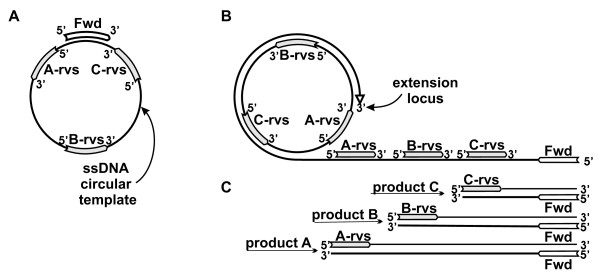
**RAM reaction diagram**. A) The circle represents a single-stranded circular DNA template molecule. A forward primer ("Fwd") is annealed to the template. "A-rvs", "B-rvs", and "C-rvs" labels refer to template sub-sequences that could serve as reverse primers. B) A RAM reaction in progress. The forward primer is displaced by its extension on the circular template to create the primary transcript that contains complementary binding sites for reverse primers. "A-rvs", "B-rvs", and "C-rvs" indicate alternate reverse-primer sequences corresponding to the template sub-sequences shown in A). The elongating primary transcript is labelled "extension locus". C) Three double-stranded products that would be formed in RAM reactions including the three reverse primers shown as examples in A) and B). The products, whose size depends on relative primer positions on the template, are formed after forward primer binding and extension on displaced extended reverse primer templates.

Figure 2A shows the key nucleic acid components of a RAM reaction: a circular ssDNA template, and forward and reverse primers. The figure depicts a forward primer annealed to a circular ssDNA template, and three possible reverse primer positions as template subsequences.

Figure 2B depicts the primer extension and strand-displacement that initiates the RAM reaction. Extension of the forward primer on the circular template displaces the 5' end of that primer; further extension creates the linear ssDNA that we designate the primary transcript. Extension of the primary transcript creates complementary binding sites for reverse primers. An extended reverse primer copies, at its 3' end, the original forward primer creating a secondary transcript.

Binding and extension of a forward primer on an extended reverse primer creates a double-stranded product whose length depends on the relative positions of the forward and reverse primers on the circular template (Figure 2B, C). We assume here without loss of generality that primer placement produces a first product that is the length of the template circle circumference, referred to here as a unit length.

### A RAM model derived from a reaction diagram

**Figure 3:**
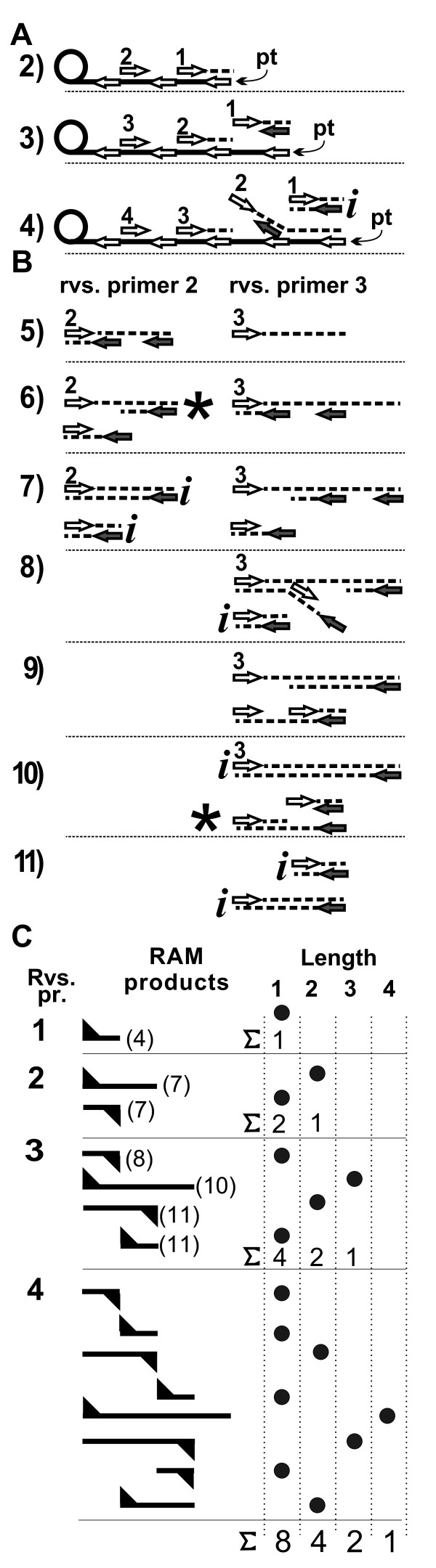
**RAM reaction process in detail**. A) Three early steps in a RAM reaction. An elongating primary transcript (pt) is shown with forward-primer sub-sequences indicated by left-pointing arrows. Reverse primers (right-pointing arrows) are numbered in their binding order. Primer extension is shown as a broken line. A slanted broken line indicates displacement of an extended primer. The letter "i" indicates an "inert" product; inert products are not shown beyond the step in which they are created. B) Fates of reverse primers 2 and 3. The circular template and primary transcript are omitted for clarity. An asterisk at reverse primer 2 product, step 6, marks a structure similar to the reverse 3 primer structure marked with an asterisk in step 10. C) Accumulation of inert RAM reaction products. The initiating reverse primer of the indicated inert dsDNA product is numbered (left column). Inert products are represented by a bar; a triangle to the bar's left indicates a product initiated by a reverse primer; a triangle to the right indicates initiation by a forward primer. The step (Figure 3, A and B) at which the product was produced is shown by a parenthesized number to the right of the reverse primer 1-3 products. Columns to the right tally the RAM reaction products by reaction step and size. Reaction products are represented by dots in length-ordered columns. For each reaction step the sum of the products produced at the current and preceding step is shown to the right of a sigma summation symbol..

Figure 3 is a diagram of the RAM reaction that leads to a quantitative description of the reaction products (expanded and annotated diagrams are in Additional File 1). The process is shown as a series of steps; a step is defined as primary transcript elongation by one template-circle circumference.

Primer extension and strand displacement produce a linear ssDNA primary transcript that is a concatamer of the complement of the circular template sequence. The first RAM-specific step is reverse primer binding to the primary transcript. (We assume that primers bind as soon as a complementary sequence is fully exposed.) As primary transcript elongation creates a second reverse-primer binding site, the first-bound reverse primer is extended to the end of the primary transcript (Figure 3A, step 2).

Figure 3A, step 3, shows the extended initial reverse primer displaced from the primary transcript by the extended second-bound reverse primer. The 3' end of the displaced strand is the complement of the forward primer; binding and extension of a forward primer results in a double-stranded product (Figure 3A, step 4). That product, and subsequently-formed double-stranded products that compose the RAM reaction product ladder, are considered inert in the sense that they do not serve further as template or primer (a test of this assumption is shown in Additional File 2).

When the second reverse primer extension product is displaced it is bound twice, successively (Figure 3A, step 4 and Figure 3B, step 5), by two forward primers. The extended product from the first-bound primer is displaced by the primer-extension product from the second-bound primer (Figure 3B, steps 5 and 6).

Figure 3B shows the extended second reverse primer completely displaced from the primary transcript at step 5. The strand orientation is such that (as on the primary transcript) strand displacement that exposes a 5' non-terminal primer-binding site allows a primer and its extension product to be displaced by extension of the adjacent primer. Reverse primer 2 will ultimately produce a double-stranded product with length equal to the circumference of the template circle plus the length of the first reverse primer product (Figure 3B, step 7).

A concise description of reverse primer 3's products can be made by noting that in Figure 3B step 10, an intermediate structure is formed (on an extended forward primer) that is similar in structure to the structure in Figure 3B, step 6 (reverse primer 2, marked by asterisks in Figure 3B) except that the positions of the forward and reverse primers are exchanged. Reverse primer 3 inert products are unit-lengths 1 (step 8) and 3 (step 10), plus products identical to reverse primer 2 products. The re-creation of functionally identical structures allows us to substitute a single description of a structure for each occurrence of that structure.

### Initial formalization of the RAM reaction model

Generalizing, the essential steps of the RAM reaction are:

1) Rolling circle amplification successively exposes new primer binding sites on the primary template.

2) Primer extension on the primary transcript releases secondary templates that are one unit longer than their immediate predecessor secondary transcript.

3) Successive primer binding and primer extension on each secondary template recursively produces one-unit-shorter tertiary templates and inert double-stranded DNA RAM products. The process terminates when the secondary template contains a single primer-binding site.

RAM product production is represented more abstractly in Figure 3C. Inert products are shown with indicators of the primer-polarity of the RAM product's first strand, with a summary count of the final reaction products from the first four reverse primers. We generalize from the diagram and the recursive model to propose that the number of molecules of a given unit-length after successive reverse-primer extensions from the primary transcript is

(1) $$N=\{\begin{array}{ll} 1, & r=p\\ 2^{r-(p+1)} & r>p \end{array};r\geq \text{p}\geq \text{1,}$$

where *N *is the number of dsDNA RAM product molecules of *p *units at the step at which reverse-primer number *r *has bound the primary transcript. (All formulae assume a single initial template molecule, and should scale linearly with template number.)

The cumulative sum of products of length p shows that the RAM reaction generates a binary series

(2) $$\Sigma N_{p}=2^{\left(r-p\right)}\text{;r}\geq \text{p}\geq \text{1;}$$

where *∑ N_p_*is the sum of products of size *p *produced from the initial reverse primer up to reverse primer *r*. That sum ideally represents the number of length *p *molecules in the reaction products, and predicts that the number of molecules per reaction product size-class should decrease by half for each step-increase in size.

Under the assumptions of the model the number *r *of reverse primers that have bound to the primary transcript is equal to (or one less than, depending on relative primer positions) the number of rounds of rolling circle amplification; using equation 2 the number of replication rounds can be computed:

(3) $$\begin{array}{l} {\text{log}}_{2}(\Sigma N_{p})=r-p\\ r=p+{\text{log}}_{2}(\Sigma N_{p}). \end{array}$$

In words, we can estimate the number of reverse-primer binding sites on the primary transcript (and thus the number of rounds of circle replication) using the number of molecules observed in a given RAM product band.

The model predicts that each reaction step adds a larger product and that the number of products of any given length doubles at each step of the reaction; and therefore that product accumulation is exponential with respect to reaction step. We presume these properties to be the basis for the log_2_(molarity) dependence on product number shown in Figure 1C. This result suggests that if the reaction goes to completion the number of molecules in each DNA band (Figure 1A) should be half the number of molecules in the preceding band.

However, as an exponential process, the RAM reaction is not expected to go to completion; and the effects of reactant-limitation or changed reaction environment on RAM product equilibrium yield and size-distribution became a question for empirical investigation. To characterize the RAM reaction in light of this model we monitored the RAM reaction in real-time, and measured RAM-product mass-distribution at various reaction time-points.

### Real-time analysis of the RAM reaction

RAM and PCR produce similar fluorescence-signal curves in real-time reactions. However, there are substantive differences between the isothermal RAM reaction and the thermocycling that is inherent to PCR. The RAM reaction is analyzed as a continuous time-dependent process rather than a discrete cycle-dependent process; the time to reach a threshold fluorescence is designated the response time (Rt) [14] corresponding to the cycle threshold (Ct) of real-time PCR. Further, in PCR the template is invariant, whereas the RAM reaction generates a dynamic collection of templates (Figure 3) as the reaction progresses.

To see whether template number affected RAM signal characteristics we measured the real-time kinetics of reactions initiated at various template levels. Because real-time instruments and analytical algorithms were developed for the PCR, we investigated whether mechanistic differences between PCR and RAM would affect real-time RAM signal analysis.

**Figure 4:**
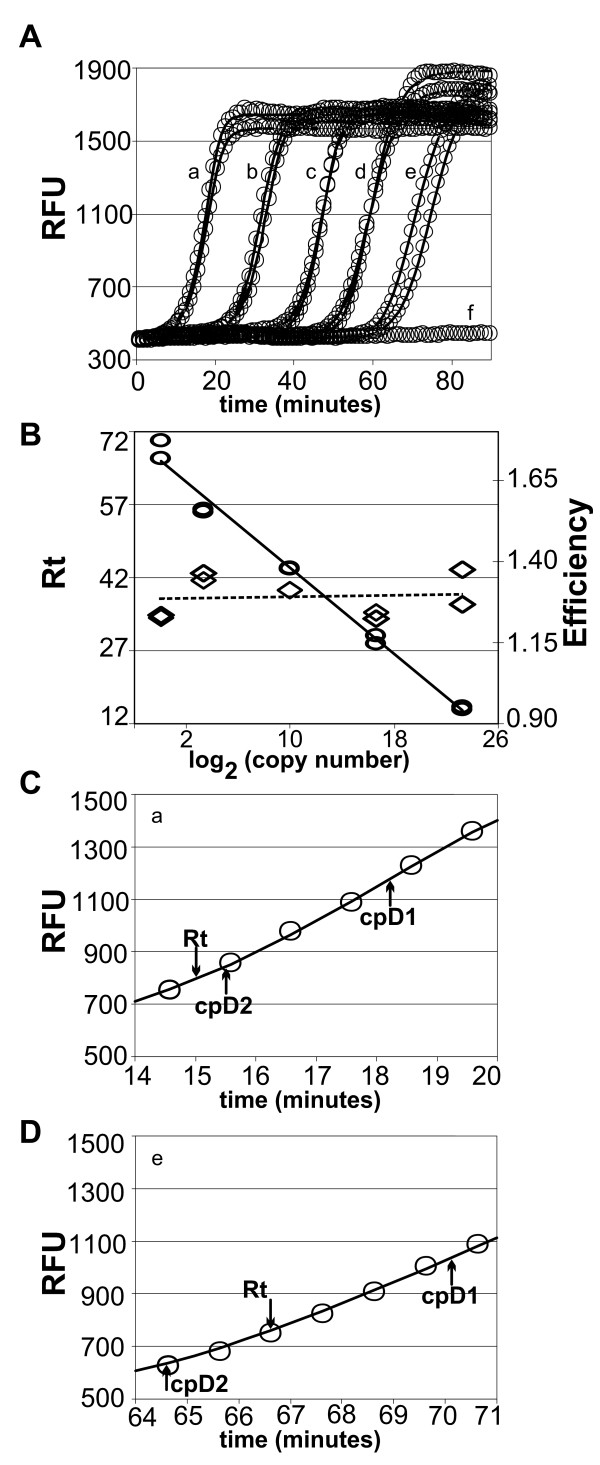
**Real-time analysis of RAM reactions**. Replicate RAM reactions were initiated with the indicated number of template molecules and fluorescent signals were collected over 15-second intervals per minute. Continuous lines through circles were generated using a model fitted to the data for each reaction. Letter-labels in Figures 4A, C, and D indicate dilution (x) followed by relative template copy number (parenthesized): a, 1 × (10^7 ^copies), b, 100 × (10^5 ^copies); c, 10^4^× (1000 copies), d, 10^6 ^× (10 copies), e, 10^7 ^× (1 copy); and f, no-template control. RFU, relative fluorescence units; Rt, response time. Figure 4A. RAM reaction discrete data points are shown as graphic circles; solid lines were generated by fitted models. Inset letters mark the template level for each pair of curves, except for no-template control signals where the data for one of four essentially identical reactions is shown. Figure 4B. Response times are plotted as ovals vs. log_2 _of the initial copy number. The solid line is a least-squares fit to the log-linear data. Diamonds indicate the reaction's efficiency; a broken line indicates a least-squares fit to the efficiency values. Figure 4C. Detail of data-points and model-curve for one of reactions "a" in Figure 4A. cpD1, maximum of first derivative; cpD2, maximum of second derivative. cpD1 marks where signal gain per unit time declines and can be taken as an upper bound of maximum efficiency amplification. Figure 4D. Detail (as for Figure 4C) of data-points and model-curve for one of reactions "e" in Figure 4A.

Figure 4A shows real-time signals generated in replicate RAM reactions on template numbers over a 10-million-fold range. Analytic decomposition of the characteristic sigmoid real-time PCR signal is an active area of research [15,16]. We used a model fitting package designed for PCR (qPCR [17,18]) to generate models for these RAM reaction data. The continuous traces shown in Figure 4A, C, and 4D were generated by the fitted sigmoid model.

Figure 4 and the correlation coefficients shown in Table 1 together suggest a reasonable fit of models to the data. Figure 4C and 4D show detail sections for the highest and lowest template levels, with response time (reported as Ct by the real-time instrument's software) and the maxima of signal vs. time first and second derivatives (cpD1, cpD2). The second derivative is frequently used to determine the Ct, and Table 1 shows that cpD2 determined by the qpcR package is in good agreement with Ct reported by the real-time instrument's software. The overall fit indicated by the correlation coefficient, cpD2 and Ct consistency, and the model fit in the signal's exponential change region together lead us to conclude that real-time RAM reactions can be analyzed using commercial or open-source software that was designed for PCR.

**Table 1:** Model fit, response time, and analysis data for a real-time RAM reaction.

Targets	10^7	10^7	10^5	10^5	1000	1000	10	10	1	1
exp.eff	1.4	1.3	1.2	1.2	1.3	1.3	1.3	1.4	1.2	1.2
cpD2	15.5	15.2	28.3	30.0	43.8	44.3	54.1	54.9	64.6	69.4
Rt	15	15.4	28.5	30.2	43.8	44	55.6	56	66.6	70.2
R^2^(adj.)	0.9993	0.9997	0.9996	0.9997	0.9995	0.9999	0.9998	0.9996	0.9997	0.9998

Legend to Table 1.

For each input template level ("Targets") the reaction efficiency (exp.eff., [17]) and second derivative maximum (cpD2) is shown, with the response time (Rt) reported (as Ct) by instrumentation software. cpD2 is the second derivative maxima of signal change vs. time; "R^2^(adj.)" is the multiple correlation coefficient describing the model's fit to the data.

Figure 4B shows the log-linear slope of Rt vs. log_2_(template copy number). (Similar plots yield the efficiency of a PCR reaction [19].) The figure shows that response time is a log-linear function of template-dilution. Table 1 (efficiency) and diamond symbols in Figure 4B show a measure of the efficiency of the reaction as determined from the fitted models; roughly equal efficiency was obtained for all reactions. Together, these data suggest that the RAM reaction rate is uncorrelated to input template number.

These observations are consistent with the RAM reaction model. At low template number, reaction time differences may result from stochastic forward primer binding time. At very high template numbers it is conceivable that primary transcript elongation may be inhibited by reactant depletion. Between those extremes dsDNA products result from secondary and tertiary transcript processing and we see no compelling reason for distinct processing modes of these transcripts as a result of input template number. However the RAM reaction rate is a complex function of several variables (unpublished data) with no a priori expectation of maximum rate, as in the PCR, and some reaction conditions may lead to template-number-sensitive reactions. However, these data suggest no necessary dependence of real-time RAM reaction kinetics on input template number.

Detailed comparison of real-time data vs. the sigmoid model via plots of residuals (Additional File 3) show that the models' deviation from the data, although small with respect to the overall signal, has a distinct time-dependent pattern. Although Figure 4A represents RAM data collected as for PCR, isothermal reactions can be monitored essentially continuously. Model fitting in PCR is required to find the exponential amplification cycles at which the Ct is ideally measured, because real-time PCR data frequently have only a few cycles of exponential amplification [16]. Continuous data collection from isothermal reactions allows simpler non-model based direct detection of exponential amplification [20].

### Characterization of a RAM-reaction time-course

The RAM model predicts that if the reaction goes to completion each product peak should have half the number of molecules as its smaller predecessor. Initial capillary electrophoresis assessment of RAM products did not show the ideal log-linear -1 slope of log_2_(molecule number) vs. product peak number. We compared product ratio slopes from RAM reaction time-course samples to determine how reaction dynamics affected deviation from the ideal slope.

**Figure 5:**
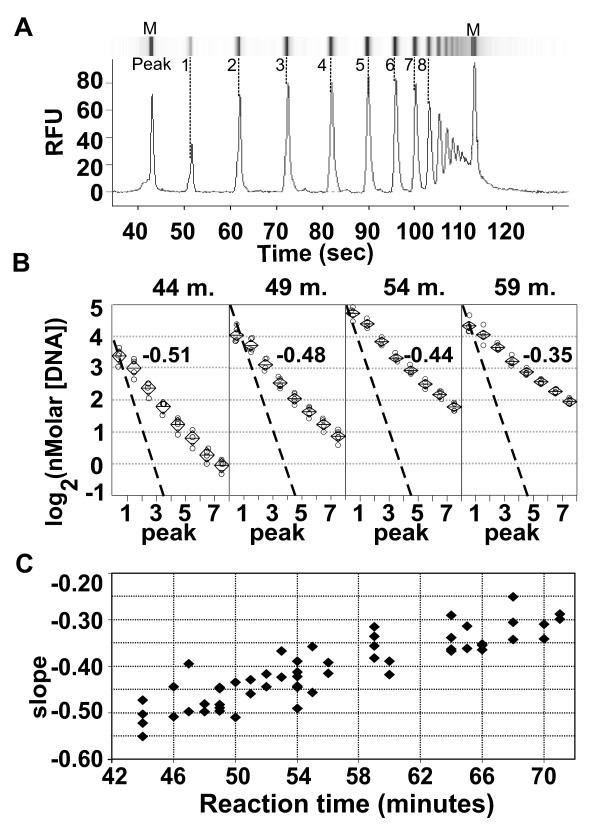
**RAM product time-course analysis**. Figure 5A: Capillary electrophoresis densitometry data with a simulated a gel-electrophoresis lane. RAM product peak numbers and capillary electrophoresis markers (M) are indicated. RFU, relative fluorescence units. Figure 5B. Per-peak DNA concentration vs. peak number (as defined in Figure 5A) is plotted for four RAM reaction time-points; reaction times (minutes) are indicated above each panel. Four independent determinations made for each time-point are shown as circles. Diamond symbols contain centered horizontal lines marking the mean for each peak and time point; the top and bottom points of the diamond mark the 95% confidence interval. Slopes determined by least-squares regression of the combined data for each time point are shown for each data group within the panels. A broken reference line showing slope -1 passes through the peak 1 data group for each time point. Figure 5C. Slope determinations are plotted for samples throughout the time course. Slopes were measured as in Figure 5B; each point represents an independent measurement.

Figure 5A shows a representative capillary electrophoresis sample of fluorescence intensity vs. time data, with a simulated gel-lane generated from the fluorescence signal. Figure 5B shows that RAM-product-ratio slopes from selected time-course samples maintain log-linearity while slope increases; Figure 5C shows slope change throughout the experiment. The slope increases (becomes less negative) with time, indicating an increasing abundance of larger molecules as the reaction proceeds.

An explanation for deviation from the ideal slope but maintaining log-linearity follows from the reaction model and stoichiometric calculation. From RAM product yield and equation 3 we infer (Additional File 1) that at least 40 to 45 reverse primers have initiated secondary transcripts to yield the products shown in Figure 4A. Complete resolution of those transcripts, under realistic conditions, would require reactant amounts that would sufficiently reduce the reactant concentration to stop the reaction (calculation details are given in Additional File 1).

The proposed model predicts that, as secondary transcripts are recursively processed into smaller tertiary transcripts, larger dsDNA products are made before smaller dsDNA products. However, smaller secondary transcripts may be fully resolved into dsDNA RAM products before reactants become limiting. Our conception of the results shown in Figure 5 is that the observed RAM products are the combination of products from fully resolved smaller secondary transcripts with larger products from partially resolved larger secondary transcripts.

One motive for the time-course experiments described above was to look for evidence that would falsify the model. In the hundreds of RAM reactions we have characterized each has had the form of the data shown in Figure 5B - a log-linear relation with negative slopes always greater than -1.

## Conclusions

This RAM model originated with an exhaustive diagram of the initial stages of the reaction, and is based on the RCA model and on the properties of a strand-displacing DNA polymerase. The utility of the Bst polymerase for the RAM reaction may lie in the balance of that enzyme's relative rate of primary transcript production and subsequent template processing. The orderly production of incrementally longer secondary transcripts may not occur if the primary transcript is produced faster than secondary transcripts can be initiated.

Our purpose is to provide a mechanistic working hypothesis for the RAM reaction. Here, we make quantitative predictions only for the theoretical reaction model; our description of the reaction products, although based on the model, is qualitative. An obvious extension of the model would be simulation of the relative contributions of completely processed vs. partially processed secondary and tertiary transcripts.

To our knowledge, this RAM reaction model is unique in its prediction of a molecular binary-power series and in invocation of a recursive molecular mechanism. These concepts are familiar from computational algorithms, and the RAM reaction may prove useful in molecular computation in addition to its current applications.

## Methods

### RAM reactions

RAM reaction time-courses were done in RAM buffer 1: 1× New England Biolabs (NEB) Thermopol II buffer, 2 mM MgSO4, 0.2 mM each dNTP; 2% DMSO; 0.75 uM forward primer Cpr4PrFwd80_20; 1 uM Cpr4PrRvs09_19; 50 molecules circularized template Cpr4Stchy_ch154 per reaction; Bst DNA polymerase, large fragment (NEB), 0.13 units/ul.

Real-time RAM reactions were done in RAM buffer 2: [1× New England Biolabs (NEB) Thermopol II buffer, 2 mM MgSO4, 0.2 mM each dNTP; 5% DMSO; 40 mM NaCl; 0.6× SYBR-Green (Molecular Probes), 1 nM fluorescein (BioRad), 0.75 uM forward primer Cpr2Fwd47_20; 1 uM Cpr3Rvs50_19; circularized template Cpr3Stchy_ch381 in the range of 0 (zero) to 10^7 ^molecules per reaction; Bst DNA polymerase, large fragment (NEB), 0.13 units/ul.

### RAM reaction time-course

A RAM reaction source-tube was assembled with all reaction components except primers and template. 40 microliters (ul) RAM reaction source was dispensed to each of 40 0.5 ml capacity reaction tubes. 10 ul of primer-template mix was added to each reaction tube, quickly mixed, then the reaction tube was held at 60°C. After incubation for the specified time (here, 33 to 72 minutes) 1 ul of 100 mM Na2EDTA was added and tubes were stored at 4°C.

### Curve-fitting to real-time data

Fluorescence vs. time data were imported as an R data frame and analyzed using the qpcR package [17]. Models were fit with the pcrbatch() function with arguments (...fct = l5(), retPar = TRUE). The parameters obtained for each reaction were used to generate the model curves at each time-point. cpD1, cpD2, experimental efficiency, and R^2^(adj) were taken from the table generated by pcrbatch().

### C-probes and primers

Cpr4Stchy_ch154 (95 DNA bases (b.)) is a padlock probe [2] that contains, 5' to 3': 25 bases of NCBI-nucleotide record AF081468 (encoding *Stachybotrys chartarum *ribosomal RNA) bases 486-510; 60 bases of internal sequence; and 10 bases of AF081468, 476-485.

Cpr4Stchy_ch154: 5'AGTATTCTCT GAGTGGCAAA CGCAAATTAA ACTCCTCCTG TGATGTCTAA TATGGTACCG

TTCGTTAGAA TCAGCTGGCA AATTCTGTTT TTTTC.

Cpr3Stchy_ch381 (100 b) is a padlock probe that contains, 5' to 3': 24 bases of NCBI-nucleotide record AF081468 (encoding *Stachybotrys chartarum *ribosomal RNA) bases 397-420; 60 bases of internal sequence; and 16 bases of AF081468, 381-396.

Cpr3Stchy_ch381: TACCTATCGT TGCTTCGGCG GGAATGAAGC TTGTCCTAGT GTGTCAGTCG CACGCTTACC

AAGAGCAACT ACACGAACAG CTGTACCCTT ATGTGAACCG.

RAM amplification primers are designated by a descriptive text prefix followed by the Cprobe base-number that initiates synthesis of the 5' complement of a forward primer on the Cprobe template, or by the first Cprobe base-number of the reverse primer subsequence; followed by an underscore separator, followed by the oligo-length.

Cpr4Fwd80_20:        5' TGCCAGCTGATTCTAACGAA

Cpr4PrRvs09_19:     5' CTGAGTGGCAAACGCAAAT

Cpr2Fwd47_23:       5' CTGACACACTAGGACAAGCTTCA

Cpr3Rvs50_20:        5' GCACGCTTACCAAGAGCAAC

### Agarose gel electrophoresis

A 300 ml, 1.2% agarose in 0.5 × TBE was cast and run in an Owl A2 electrophoresis chamber. 4 ul RAM reaction (58 minute sample from the time course described above) and 10 ul BRL 1 kb ladder were loaded to the wells of the gel lanes shown in Figure 1. The gel was run in 0.5× TBE running buffer for 200 minutes at 180 Vdc., stained in 1× SYBR-Gold (Molecular Probes) in 0.5 × TBE for 20 minutes, then imaged on a Spectroline model TI-312E UV-transilluminator through a Wratten No. 15 filter (sold by Molecular Probes as a SYBR Green/Gold photographic filter).

The gel image was captured with a Nikon 990 (E990V1.0) digital camera in aperture-priority mode; F6.0 at effective focal length f13.7 mm, 5.10 second exposure, "FULL FINE" mode. All digital manipulations were applied uniformly until separation of selected lanes for image assembly. The image was rotated to vertical orientation, reversed to create a black-on-white image; then contrast and brightness were adjusted. The chosen lanes were cropped and placed in the final figure.

### Capillary electrophoresis (CE)

DNA mass and size measurements were done in an Agilent Bioanalyzer 2100 and analyzed with software version B02.06 SI 418 Patch level 01. DNA1000 (Series II) assays were performed according to the manufacturer's protocol. 1 ul RAM reaction mix was loaded to each CE chamber.

CE data processing: Electropherogram representations of the data were reviewed to correct occasional incorrect assignment of electrophoretic peak to internal markers. The data was exported as text files for further analysis. RAM products were assigned peak numbers, designating the lowest molecular weight product as peak 1. Slopes were determined by linear regression of log_2_(nMolar DNA) vs. peak number for the first 8 RAM product peaks.

Analysis of CE data: Figure 4B data panels were constructed in JMP (SAS Institute, Version 4.0.4) as a oneway analysis of variance of log_2_(nMolar concentration) vs. peak number for each time point shown. Figure 4C shows slope (determined as described above) for each reaction containing sufficient DNA for detection in the Agilent instrument.

## Competing interests

Thorne Diagnostics, Inc. employs the authors and paid page charges. Thorne Diagnostics is developing commercial assays using rolling circle amplification under license. The content of the manuscript is not the subject of any pending patent application and the authors do not stand to gain or lose financially as a result of publication.

## Authors' contributions

TPB conceived the RAM reaction model, performed experiments and analysis, and wrote the manuscript. JHS and RMN performed experiments and reviewed the manuscript. DJL provided constructive criticism and discussion during development of the model. All authors read and approved the final manuscript.

## Supplementary Material

Additional file 1 **Expanded and annotated diagram of **Figure 2A**(early steps in the RAM reaction), and extension of the model for product prediction**. Figure S1.1 expands on Figure 3, showing generation of RAM products by primer extension on a template generated by rolling circle amplification. Figure S1.2 illustrates the recursive, or nested, model of RAM products. Tables S1.1, S1.2, and S1.3 extend the table of products concept to generate a prediction of exponential dsDNA product accumulation shown in Figure S1.3. Table S1.4 shows an example of RAM stoichiometry calculation, followed by data and arguments to show that RAM reactions as performed could exhaust the reagent pool. Click here for file

Additional file 2 **Experimental test of the assumption that RAM products are inert in the RAM reaction**. Click here for file

Additional file 3 **Residuals of fitted real-time model**. Click here for file
