# Supplementary material for: A mechanism for ramified rolling circle amplification
Source: BMC Mol Biol. 2010 Dec 7;11:94. doi: 10.1186/1471-2199-11-94 (PMC3017024; doi:10.1186/1471-2199-11-94)
Supplement: Additional file 1 — Expanded and annotated diagram of Figure 2A(early steps in the RAM reaction), and extension of the model for product prediction. Figure S1.1 expands on Figure 3, showing generation of RAM products by primer extension on a template generated by rolling circle amplification. Figure S1.2 illustrates the recursive, or nested, model of RAM products. Tables S1.1, S1.2, and S1.3 extend the table of products concept to generate a prediction of exponential dsDNA product accumulation shown in Figure S1.3. Table S1.4 shows an example of RAM stoichiometry calculation, followed by data and arguments to show that RAM reactions as performed could exhaust the reagent pool. [file 1471-2199-11-94-S1.PDF]

Beals, Smith, Nietupski, and Lane

Supplementary materials 1

Figure S1.1 is an enlarged and annotated version of Figure 3A and B. The figure shows binding, extension, and resolution into dsDNA products for the first 3 reaction steps. Figure 3B and Figure S1.1 show that (as noted in the text) among reverse primer 3's products are the structural equivalents of reverse primer 2's products.

Figure S1.2 is an *aufbau* ("building up") diagram showing how each successive secondary transcript that is released from the primary transcript incorporates into its products a structurally similar copy of all prior secondary transcripts' products. Beginning with secondary transcript 3 each secondary transcript's incorporation of structurally equivalent products is illustrated by a colored background.

Column "primers" in S1.2 records for each secondary transcript the number of forward and reverse primer pairs that will be incorporated in its inert dsDNA products. Each inert product incorporates one forward and one reverse primer so the number of primers consumed, either forward or reverse, is equal to the number of inert products. Column "∑ primers" records the cumulative sum of primers used.

The rate of the reaction, as measured e.g. in real time, is the rate of accumulation of dsDNA inert products. To make quantitative predictions of dsDNA mass accumulation, Figure S1.2 column labeled "unit lengths" records for each step the sum of lengths of products produced in that step, and we observe that product to be numerically equal to the "∑ primers" column. That numerical equivalence appears to be the result of the incorporation of all predecessor products among each transcripts' products.

Finally, column "∑ unit lengths" is the cumulative sum of "unit lengths". Column "∑ unit lengths" records the dsDNA mass accumulated in the current and earlier reaction steps. That accumulated dsDNA mass is what is detected in e.g. a real-time reaction.

Fate of reverse primer number...

Step		1	2	3
1	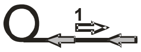	binds primary template (p.t.)		
2	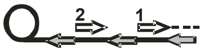	extended 1 unit	binds p.t.	
3	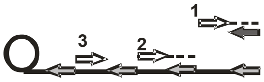	displaced; bound by a forward primer	extended 1 unit; displaces reverse primer 1	binds p.t.
4	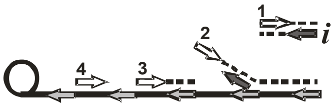	made double-stranded, inert	extended 2 units; half - displaced; bound (non-term) by forward primer	extended 1 unit; begins to displace reverse primer 2

Step		**primer 2's fate**	**primer 3's fate**
5	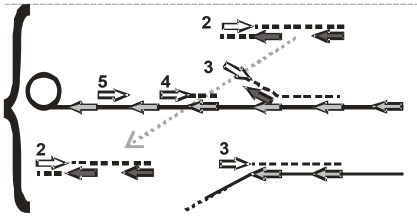	displaced; first forward primer extends 1 unit; bound by second forward primer	extended 2 units; half-displaced by reverse primer 4; bound by non-terminal forward primer
6	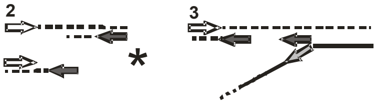	second forward primer extends 1 unit; displaces first forward primer. *, see primer 3, step 10.	extended 3 units; 2 units displaced; bound by another non-terminal forward primer; first forward primer extended 1 unit
7	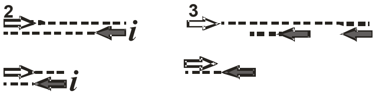	second forward primer full extend, inert; first forward primer's reverse primer extends; inert.	Fully displaced; bound by terminal forward primer. Extension of second-bound forward primer displaces first-bound forward primer; displaced first-bound forward primer bound by reverse primer.

Step		**primer 3's fate**
8	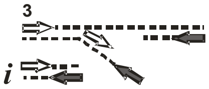	2cd bound forward primer displaced by terminal forward primer extension and binds a reverse primer. First-bound forward primer made inert.
9	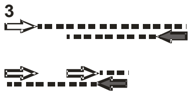	2cd bound forward primer structure like reverse primer 2 step 5. Terminal forward primer extended 2 units.
10	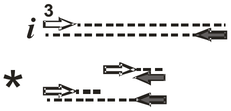	Terminal forward primer extended 3 units to form inert product. *, see primer 2, step 6.
11	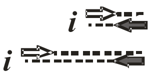	Last of reverse primer 3 products made inert.

Figure S1.1

Beals, Smith, Nietupski, and Lane

Supplementary material 1

		Product length								summary counts			
Step #	Step row	1	2	3	4	5	6	7		primers	∑ primers	unit lengths	∑ unit lengths
1	1	1								1	1	1	1
2	1	1	2							2	3	3	4
3	1	1		3						4	7	7	11
	2	1	2										
4	1	1			4					8	15	15	26
	2	1	2										
	3	1		3									
	4	1	2										
5	1	1				5				16	31	31	57
	2	1	2										
	3	1		3									
	4	1	2										
	5	1			4								
	6	1	2										
	7	1		3									
	8	1	2										
6	1	1					6			32	63	63	120
	2	1	2										
	3	1		3									
	4	1	2										
	5	1			4								
	6	1	2										
	7	1		3									
	8	1	2										
	9	1				5							
	10	1	2										
	11	1		3									
	12	1	2										
	13	1			4								
	14	1	2										
	15	1		3									
	16	1	2										
7	1	1						7		64	127	127	247
	2	1	2										
	3	1		3									
	4	1	2										
	5	1			4								
	6	1	2										
	7	1		3									
	8	1	2										
	9	1				5							
	10	1	2										
	11	1		3									
	12	1	2										
	13	1			4								
	14	1	2										
	15	1		3									
	16	1	2										
	17	1					6						
	18	1	2										
	19	1		3									
	20	1	2										
	21	1			4								
	22	1	2										
	23	1		3									
	24	1	2										
	25	1				5							
	26	1	2										
	27	1		3									
	28	1	2										
	29	1			4								
	30	1	2										
	31	1		3									
	32	1	2										

Figure S1.2. *Aufbau* diagram of RAM products.

We want to make predictions about reactant requirements, product number; and product mass per step in a compact and formulaic form. We begin with another table, with one row per reaction step. For each reaction step (numbered by reverse primer as before) we record the number of products of unit length – we are not (as in Figure S1.2) recording the product length in this table. We fill the table using equation 1 from the text:

rvs-pr *r*	unit-length *p* 1	2	3	4	5	6
1	1					
2	1	1				
3	2	1	1			
4	4	2	1	1		
5	8	4	2	1	1	
6	16	8	4	2	1	1

Table S1.1. RAM product counts.

if (***r*** = ***p***,
  1,
  else
  2^(***r*** - (***p*** +1))
)

and observe that the table is consistent with a count of products per step in Figure S1.2.

To this table we now add a set of columns as in Figure S1.2. The reverse primer/reaction-step column remains to the left, and we insert columns for counting primers/products; cumulative primers/products; unit lengths, and cumulative unit lengths.

rvs. pr. *r*	primers	Σ primers	unit lengths	Σ unit lengths	unit-length *p* 1	2	3	4	5	6
1	1	1	1	1	1					
2	2	3	3	4	1	1				
3	4	7	7	11	2	1	1			
4	8	15	15	26	4	2	1	1		
5	16	31	31	57	8	4	2	1	1	
6	32	63	63	120	16	8	4	2	1	1

Table S1.2. RAM product counts with summary columns.

Column "primers" counts the equivalent number of inert dsDNA products without considering the products length or mass. Product number doubles per step and is numerically equal to 2^(***r***-1) - the sum 1, 2, 4, … The cumulative sum in column "∑ primers" of primer/product number in the interval [1,***r***] is the geometric sum $\sum_{k=1}^{r} 2^k$.

As described for Figure S1.2, product mass at step ***r*** is numerically equal to the cumulative sum of primer/product number in the interval [1,***r***]. We computed the cumulative sum by dsDNA mass = 2^(***r*** + 1) - (***r*** +2) and checked this empirical formula vs. sums calculated in the full table. We can now construct a table (Table S1.3) of products that can be continued to arbitrary ***r*,** separate from the expanding table of predicted products.

rvs. pr. *r*	primers / products	∑ products	unit lengths	∑ lengths
1	1	1	1	1
2	2	3	3	4
3	4	7	7	11
4	8	15	15	26
5	16	31	31	57
6	32	63	63	120
⋮	⋮	⋮	⋮	⋮
r	2^(*r*-1)	(2^*r*)-1	(2^*r*)-1	(2^(*r* + 1)) - (*r* + 2)

Table S1.3. RAM product summary columns with formulae.

Plotting log(cumulative product mass) vs. reaction step yields Figure S1.3, showing exponential accumulation of dsDNA per reaction step (again, steps are numbered as reverse primer number). The model's deviation from log-linearity at low reverse template numbers results from the "-(r+2)" term for reverse-primers 1 and 2, a level well below current laboratory-observable product detection.

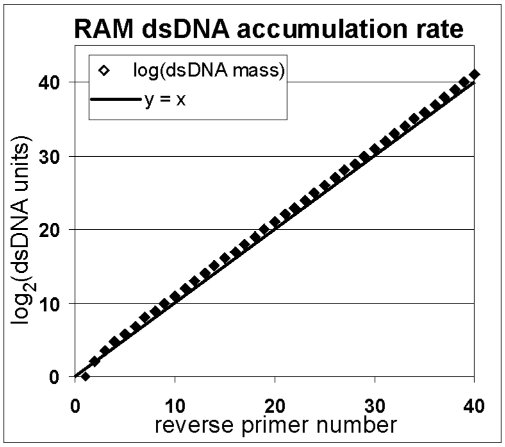

Figure S1.3. RAM product accumulation vs. reaction step (reverse primer number).

We now use Table S1.3 and formulae for stoichiometric calculation.

<u>Primers as reagents</u>

Each product requires a forward and a reverse primer. Suppose we use 0.75 micromolar (uM) primers in a 50 microliter (ul) reaction (as for the forward primer in the RAM reaction time-series described in the text). Then we are adding

50 ul is 5.0E-05 liters
0.75 uM is 7.5E-07 molar
5.0E-05 liters * 7.5E-07 moles / liter = 3.8E-11 moles
3.8E-11 moles * 6.0E+23 molecules/mol = 2.3E+13 molecules.
$\log_2$(molecules) = 44.4

From Figure S1.2 or Tables S1.2, S1.3 we see the cumulative number of primers/products per reaction step is 2^(step number) -1. Table S1.4 shows that after the first few reaction steps, $\log_2$(primer molecule number) shows the **approximate** number of reaction steps that primer number can serve (ignoring required primer concentration). These calculations are for a single template molecule; for multiple template molecules, divide the primer number by template molecules; in log-space, subtract $\log_2$(template number).

rvs. pr. *r*	primers / products	Σ products	log2 (Σ pr.)	*r* - log2 (Σ pr.)
1	1	1	0	1
2	2	3	1.58	0.42
3	4	7	2.81	0.19
4	8	15	3.91	0.09
⋮	⋮	⋮	⋮	⋮
8	128	255	7.99	0.01
⋮	⋮	⋮	⋮	⋮
12	2048	4095	12.00	3.5E-04

Table S1.4. Log2(primer number) approximates reaction step.

From the calculations above, $\log_2$(primer molecules) < 45. That is, for a single template circle, a primary transcript that is 45 units long would exhaust the primers added to the above reaction. Of course, the actual reaction will pause when any reactant's concentration drops below its minimum.

Estimating RAM reaction step number (primary transcript length)

We calculate an estimate of the number of rounds of rolling circle replication using as an example the RAM products illustrated in Figure 4. As noted in the text, the slope ($\log_2$ molar [DNA] vs. peak number) is greater than –1 and increases with reaction time.

If the slope had been the ideal –1 we would calculate the same circle number for each peak. But there is a greater proportion of larger molecules than would be seen in an ideal reaction, so the unit-number-length of primary transcripts will be different when calculated from different peaks. We calculate an estimate of the primary transcript length as follows.

For time point 54 (Figure 4B) the capillary electrophoresis instrument calculates the concentration of the peak 1 product to be 25.5 nanomolar.

25.5 nanomoles / liter * 1.0E-09 mol/nmol = 2.6E-08 moles / liter

50 ul reactions are 5.0E-05 liters

2.6E-08 moles / liter * 5.0E-05 liters = 1.3E-12 moles

1.3E-12 moles * 6.02E+23 molecules/mol = 7.7E+11 molecules

7.7E+11 molecules / 50 templates = 1.5E+10 molecules/input template.

Using equation 3 from the text we calculate number of reverse primers and estimate primary transcript length:

r = 1 (peak 1) + log2(1.5E+10) = 34.5.

Similar calculations for peak 8 (3.8 nanomolar) finds 2.3E+9 molecules/template, and

r = 8 (peak 8) + log2(2.3E+9) = 39.1.

50 templates were used in these reactions; log2(50) = 5.6

Scale primers-capacity (calculated above) for template number: 44.4 – 5.6 = 38.8

The calculated primary transcript length (39.1) is greater than the primer-capacity (38.8) at the template number used. If the reaction went to completion it would exhaust all the primers that were added to the reaction. Of course, the actual reaction will pause when any reactant's concentration drops below a minimum.
