# Supplementary material for: A mechanism for ramified rolling circle amplification
Source: BMC Mol Biol. 2010 Dec 7;11:94. doi: 10.1186/1471-2199-11-94 (PMC3017024; doi:10.1186/1471-2199-11-94)
Supplement: Additional file 2 — Experimental test of the assumption that RAM products are inert in the RAM reaction. [file 1471-2199-11-94-S2.PDF]

Supplementary material 2: Experimental test of the assumption that double-stranded RAM products are inert in the RAM reaction.

Our RAM reaction model assumes that double-stranded RAM products are an inert component of the RAM reaction. To test the reactivity of RAM products in the RAM reaction, an aliquot of a RAM reaction was exposed either to S1 single-strand-specific endonuclease or to mock-S1 conditions (S1-buffer without enzyme). After incubation as recommended by the enzyme manufacturer (Promega) the S1 and mock reactions were extracted once with phenol:chloroform:isoamyl alcohol then nucleic acids were recovered from a Qiagen PCR purification matrix. Recovered materials were visualized and the yield was estimated on an Agilent Bioanalyzer 2100 (Figure S2.1).

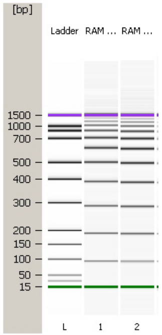

Figure S2.1. Capillary electrophoresis profiles. Lane 1, mock; lane 2, S1 treated.

A dilution series of the S1-treated RAM products was made such that the number of molecules of the smallest RAM product (the first peak on the Agilent electrophoretic trace, peak 1) was in the same range as the number of single-stranded circles that can be conveniently assayed. Specifically, S1-treated RAM products were diluted such that between 50 and 5x10^7 peak1 molecules could be added to a RAM reaction.

A dilution series of the same lot of circle-template that had been used to generate the RAM product was prepared such that between 50 and 10^5 circles could be added to the RAM reaction. Master mixes were prepared and RAM reactions were assembled such that each template-type (single-stranded circular DNA molecules or RAM products) was combined with aliquots of the same reaction buffer and primers. All RAM reactions were run as triplicates (S1 RAM product template) or as quadruplicates (single-stranded DNA circles) in a single 96-well reaction plate; assignment of reaction-type to well was randomized throughout the reaction plate.

level	count	fraction fail	Poisson point est.	level / Poisson pnt. est.
5	3	1		
50	3	1		
1000	3	2/3	0.41	2466
5000	3	1/3	1.10	4551
500000	3	0		
50000000	3	0		

Table S2.1. Failure rate for RAM reactions on RAM product templates. Poisson point estimates are -ln(fraction failed)[1].

Preliminary inspection of fluorescent signal-traces from the real-time reaction showed that the per-molecule response-time of single-stranded circular DNA templates was faster than the per-molecule response-time of S1 RAM product templates. The minimum number of RAM products required to reliably initiate a RAM reaction can be crudely estimated to be greater than 2000 molecules (Poisson point estimate[1] in Table S2.1).

Regression-lines of response-time vs. $\log_2$(input molecule) were fit for both template types (Figure S2.2). An amplification-efficiency-ratio was calculated for single-stranded DNA circles vs. S1 RAM product template. The response-time for each S1 RAM product template level was used to calculate the number of single-stranded DNA circles that would be required to produce that response time. The ratio of the predicted number of circles divided by the calculated number of input S1 RAM product peak-1 molecules was taken as the amplification efficiency.

Over all input molecule-ranges, the amplification efficiency ratio of RAM-products to circles was in the less than 0.05 percent range; that is, it took greater than 2000 peak-1 S1-treated RAM products to produce the same signal as one single-stranded DNA circle. This is probably an over-estimate of the amplification potential of S1-treated RAM products, because only the peak1 molecules were used to estimate RAM product molecule number. The Poisson failure estimate and the circle-molecule ratio comparisons both depend on the estimate of RAM product molecules number, but are otherwise independent.

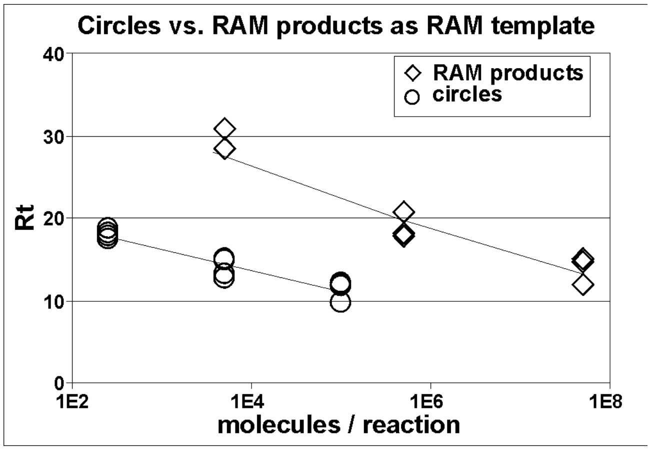

Figure S2.2. Log-linear plot of response-times for circles vs. RAM products as RAM reaction templates.

We consider these results to be consistent with the idealized assumption made in the RAM reaction model, that is, that double-stranded RAM products are inert in the RAM reaction.

[1] *The Design of Experiments*, R.A. Fisher, p. 218; Oxford University Press, New York. © 1990
