# Supplementary material for: A mechanism for ramified rolling circle amplification
Source: BMC Mol Biol. 2010 Dec 7;11:94. doi: 10.1186/1471-2199-11-94 (PMC3017024; doi:10.1186/1471-2199-11-94)
Supplement: Additional file 3 — Residuals of fitted real-time model. [file 1471-2199-11-94-S3.PDF]

Residuals of models vs. real-time data.

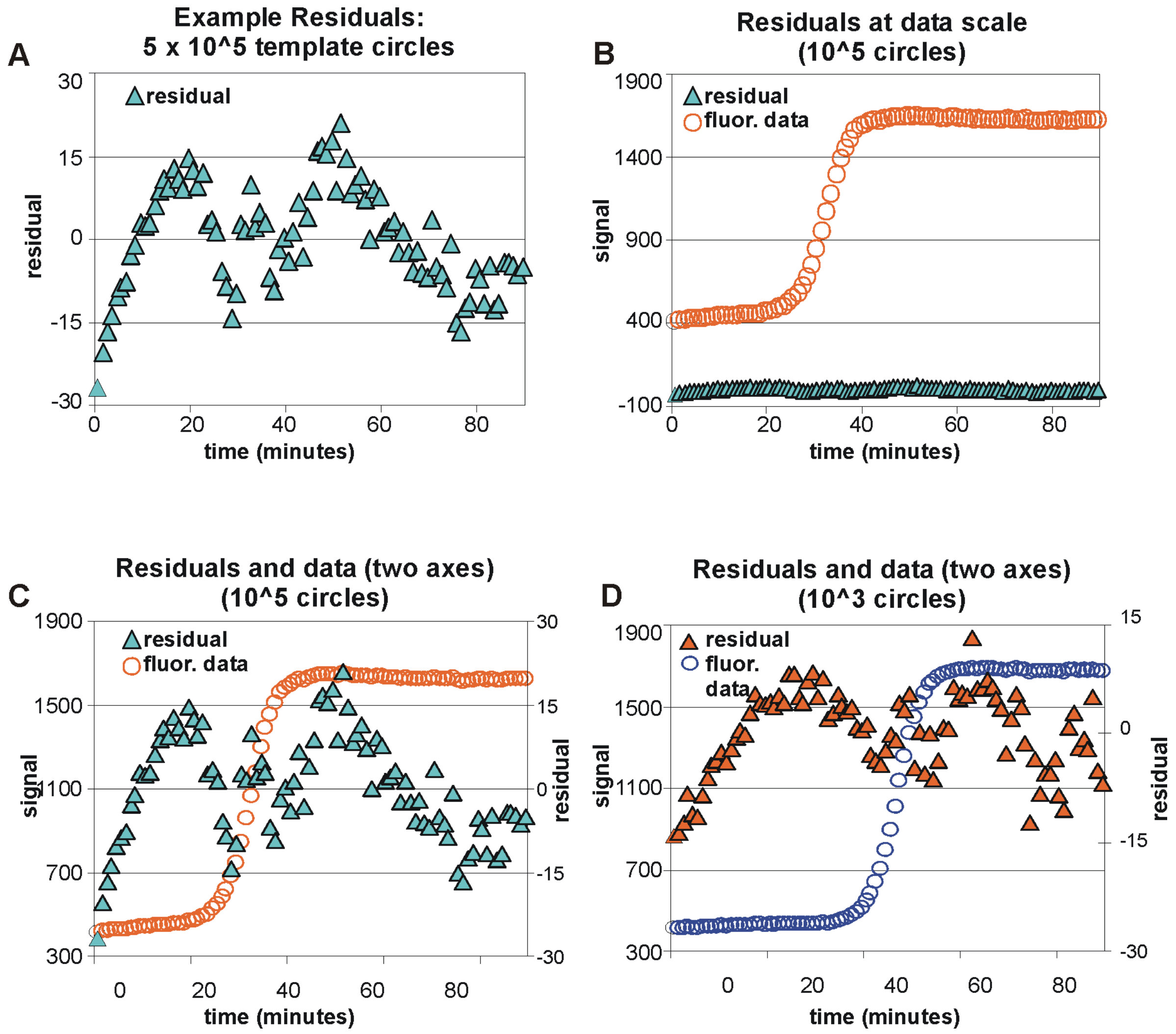

Figure S3.1

Figure S3 plots the differences ("residuals") observed vs. model-predicted fluorescent signal, calculated as (observed – model); the residuals appear to be non-random with respect to time.

Figure S3.1A shows residuals vs. reaction time. The data are from one of the 10^5 copy-number RAM reactions shown in Figure 4A.

Figure S3.1B shows that the magnitude of the residual is small compared to the overall magnitude of the change in fluorescence over the whole reaction.

Figure S3.1C combines Figures S3.1A and S3.1B, with the magnitude for the residuals shown on the right axis; the magnitude of the residuals can be interpreted at the various points of the amplification reaction: baseline deviation, exponential growth, plateau.

Figure S3.1D shows results similar to Figure Sx.1C for an independent reaction with different initial template number.
